# Does financial inclusion improve the welfare of people living with disabilities? evidence from Indonesia using a propensity score matching approach

**DOI:** 10.1186/s12889-026-27080-2

**Published:** 2026-03-20

**Authors:** Muhammad Anif Afandi, Adiatma Yudistira Manogar Siregar, Maman Setiawan, Nury Effendi

**Affiliations:** 1 Doctoral Program of Economics, Faculty of Economics and Business, Universitas Padjadjaran, Bandung, Indonesia; 2 Department of Banking and Finance, Universitas Muhammadiyah Metro, Kota Metro, Lampung Indonesia; 3 Center for Economics and Development Studies, Department of Economics, Faculty of Economics and Business, Universitas Padjadjaran, Bandung, Indonesia

**Keywords:** Financial inclusion, Welfare, People living with disabilities, Propensity score matching

## Abstract

**Background:**

People living with disabilities have the same rights as non-disabled individuals to access financial services. However, in 2023, as many as 75.7% of people living with disabilities in Indonesia still did not own a bank account, even though account ownership is the most fundamental element of financial inclusion. This study aims to analyze the association between financial inclusion on the welfare of people living with disabilities in Indonesia.

**Methods:**

This study employed cross-sectional data from the March 2023 National Socio-Economic Survey (Susenas) to analyze the association between financial inclusion on the welfare of people living with disabilities, focusing on household heads with disabilities as the unit of analysis, representing a weighted sample of 8,726,103 households. Welfare is proxied by the average household expenditure of the household head, which includes both food and non-food components such as health and education spending. The Propensity Score Matching (PSM) technique was applied using a probit-based model via Stata software. The assumptions of common support and standardized bias were verified and satisfied.

**Results:**

Using three different financial inclusion index cut-off points—≥25%, ≥ 50%, and ≥ 75%—the results revealed that the Average Treatment Effect on the Treated (ATT) of financial inclusion positively and significantly associated with the welfare level of household heads with disabilities, ranging from 22.83% to 23.43%, 29.47%-29.87%, and 31.23%-33.23%, respectively. These findings indicated that higher levels of financial inclusion among household heads with disabilities were associated with higher levels of welfare attainment.

**Conclusion:**

The findings suggest that financial inclusion programs play a crucial role in enhancing the welfare of people living with disabilities, as they have a positive and significant association on improving their well-being.

**Supplementary Information:**

The online version contains supplementary material available at 10.1186/s12889-026-27080-2.

## Background

Recent global economic disruptions have underscored the persistent vulnerability of populations at the lower end of the socioeconomic spectrum, particularly socially marginalized groups. The COVID-19 pandemic, in particular, has disproportionately affected people with disabilities, who are historically socially vulnerable—not inherently vulnerable—due to structural exclusion, limited social participation, and constrained access to economic and institutional resources. Public health measures implemented to contain the pandemic, such as lockdowns and enforced quarantines, while necessary, have often exacerbated pre-existing disadvantages when not carefully designed, resulting in adverse educational, occupational, and socio-economic consequences for people living with disabilities. These conditions have further limited their financial buffers and access to formal financial services, thereby increasing the risk of widening health and socio-economic disparities [1, 2]. In response to these contemporary challenges, financial inclusion has become a core component of the global development agenda, emphasizing expanded access to formal and digital financial services as a mechanism for enhancing household resilience and mitigating welfare deterioration, especially in developing economies where financial exclusion remains widespread [3].

Within this context, people living with disabilities constitute a key population whose rights to equitable access to financial services must be explicitly addressed. Globally, people living with disabilities account for approximately 16% of the world’s population, or about 1.3 billion people [4]. In developing countries such as Indonesia, the number of people living with disabilities is estimated at 7.7% of the total population, or around 17.8 million individuals in 2022 [5]. Alarmingly, 75.7% of people living with disabilities in Indonesia did not own a bank account in 2023, even though bank account ownership constitutes a fundamental element of financial inclusion [5].

To address this issue, the Indonesian government has undertaken various initiatives to promote financial inclusion. One major step was the formulation of the National Strategy for Financial Inclusion (SNKI) in 2012. In 2016, the government reinforced this commitment through Presidential Regulation No. 82/2016 on SNKI, which was later amended by Presidential Regulation No. 114/2020, explicitly including people living with disabilities as one of the target groups of the national financial inclusion agenda [6]. Furthermore, Article 19 [2](d) of the Financial Services Authority Regulation No. 3/2023 mandates financial service providers to provide special facilities to enhance financial inclusion for people living with disabilities [7].

Despite these policy measures, people living with disabilities are widely recognized as a vulnerable group facing multidimensional poverty [8, 9]. Although the national employment rate among people living with disabilities in Indonesia reached 56.98%, approximately 71.4% of them are engaged in the informal sector—primarily in agriculture, forestry, and fisheries—where they experience unstable income and lack access to employment protection and social security [10].

In line with this concern, a growing body of literature has shown that financial inclusion can reduce poverty and inequality by enabling individuals and households to perform financial transactions, invest, manage risks, and access credit from formal institutions [11–13]. In the context of people living with disabilities, financial inclusion—such as bank account ownership—also functions as a gateway for receiving government assistance, which is essential given their heightened vulnerability to multidimensional poverty [10].

Furthermore, previous empirical studies have consistently found that financially included household heads tend to enjoy higher welfare levels compared to their financially excluded counterparts [14–20]. However, most of these studies have focused on the general population and have not specifically examined the association of financial inclusion on the welfare of people living with disabilities. In Indonesia, empirical studies addressing this issue remain limited. Therefore, this study thus seeks to fill this research gap, representing the novel contribution of the present analysis.

At the same time, welfare disparities between people living with and without disabilities remain evident in Indonesia. In 2020, the poverty rate among people living with disabilities stood at 11.42%, compared to 9.63% among non-disabled individuals. The rate was even higher—13.38%—among individuals with multiple disabilities [10]. Given these persistent inequalities and the pivotal role of financial inclusion in promoting economic development, this study examines the association of financial inclusion on the welfare of people living with disabilities in Indonesia, with welfare defined specifically in its financial dimension and proxied by average household head expenditure. To enhance clarity, this study pursues the following specific aims:

- First, to examine the quasi-causal association between financial inclusion and the financial welfare of people living with disabilities in Indonesia, using a composite financial inclusion index constructed from multiple indicators.
- Second, to assess whether the welfare associated with financial inclusion differ across varying degrees of inclusion by applying alternative financial inclusion cut-off points.
- Third, to provide empirical evidence that can inform evidence-based and inclusive financial policies aimed at improving the welfare of people living with disabilities in Indonesia.

By explicitly focusing on the financial dimension of welfare and on people living with disabilities—an under-researched population in Indonesia and Southeast Asia—this study contributes to the existing literature and offers policy-relevant insights to support disability-inclusive financial development.

## Methods

### Study design and data source

This study employed a cross-sectional observational study design using secondary data derived from the National Socio-Economic Survey (Survei Sosial Ekonomi Nasional—Susenas), a large-scale nationally representative survey conducted regularly by Statistics Indonesia (Badan Pusat Statistik/BPS). Specifically, this study utilized the March 2023 wave of Susenas. The data are publicly accessible upon formal request through the official BPS data portal https://silastik.bps.go.id/v3/index.php/site/login/. The unit of analysis in this study is household heads with disabilities. The analysis focuses on examining the association of financial inclusion on welfare, proxied by average household head expenditure, among people living with disabilities in Indonesia. The reporting of this study follows the Strengthening the Reporting of Observational Studies in Epidemiology (STROBE) guidelines.

In this study, people living with disabilities are defined based on the official definition used by Statistics Indonesia (BPS) and the Indonesian Law No. 8 of 2016. Disability refers to long-term physical, intellectual, mental, and/or sensory impairments which, in interaction with environmental barriers, may hinder full and effective participation in society on an equal basis with others. Accordingly, the disability measures in the Susenas survey capture long-term functional difficulties rather than temporary limitations due to short-term illness or accidents. The disability classification in Susenas is primarily based on the Washington Group Short Set (WGSS) of questions, a widely used and publicly available universal disability assessment tool designed to identify functional difficulties across core domains of daily functioning. In addition to WGSS domains, Susenas includes several nationally adapted functional indicators to reflect the Indonesian context. Accordingly, disabilities are identified based on various functional difficulties, including visual impairment, hearing impairment, mobility difficulties (walking or climbing stairs), hand or finger movement limitations, memory or concentration difficulties, behavioral or emotional disorders, speech or communication impairments, and challenges in self-care. Each type of difficulty is categorized into three levels of severity: some difficulty, a lot of difficulty, and cannot perform the activity at all [21].

## Sample selection and sample size

The sampling process in this study followed the official design of the National Socio-Economic Survey (Susenas), which employs a stratified multistage sampling framework to produce nationally representative estimates [22]. The initial dataset from the March 2023 Susenas wave recorded 72,891,604 household heads at the population level after applying sampling weights.

In line with the focus of this study, the analytical sample was restricted to household heads living with disabilities. Disability status was identified using the functional difficulty indicators collected in Susenas. Based on this criterion, 8,726,103 household heads (11.97% of the total weighted population) were classified as having disabilities, while 64,165,501 household heads (88.03%) without disabilities were excluded from the analysis.

The Susenas dataset applies sampling weights with complex adjustments to ensure population-representative estimates [22]. The unweighted analytical sample consists of 44,107 household heads with disabilities, which represents an estimated population of 8,726,103 household heads with disabilities at the national level after applying the official sampling weights provided by Statistics Indonesia (BPS). All subsequent analyses in this study are conducted using weighted data to ensure nationally representative results.

Within the weighted population of household heads with disabilities, the majority experienced varying degrees of functional limitations. Specifically, 6,801,342 individuals (77.99%) reported mild difficulties or limitations, 1,338,450 individuals (15.33%) reported moderate difficulties, and 586,311 individuals (6.72%) were classified as having severe disabilities or being unable to perform daily activities.

### Statistical analysis

#### Measurement of the financial inclusion index

Financial inclusion is broadly defined as adequate access to useful and affordable financial products and services that meet diverse financial needs—such as transactions and payments, savings, credit, and insurance [23, 24]. Based on this conceptual framework, the present study adopts four dimensions of financial inclusion, namely [1] ownership of a savings account [2], use of financial products or services [3], access to credit, and [4] ownership of health insurance.

These four dimensions form the basis of a composite financial inclusion index, with each indicator assigned an equal weight of one, resulting in an index ranging from 0 to 4. This index captures both the breadth and intensity of engagement with formal financial services. To facilitate causal comparison within a propensity score matching framework, the index is subsequently transformed into a binary treatment variable.

The primary cut-off point is set at ≥ 50%, corresponding to access to at least two out of the four financial inclusion dimensions. This threshold reflects a meaningful level of financial engagement beyond minimal or incidental access and has been widely adopted in previous studies [14, 15, 19]. Accordingly, household heads with disabilities who score 2–4 are classified as financially included, while those scoring 0–1 are classified as financially excluded.

To address concerns regarding potential oversimplification of this multidimensional construct and to assess the robustness of the findings, additional analyses are conducted using alternative cut-off points of ≥ 25% (at least one indicator) and ≥ 75% (at least three indicators). These alternative thresholds capture varying degrees of financial inclusion intensity and allow us to examine whether the estimated associations with welfare outcomes remain consistent across different levels of financial engagement.

#### Propensity Score Matching (PSM)

Propensity score refers to the probability that an individual receives a particular treatment in an observational study, given a set of observed characteristics (covariates) [25]. This score serves as the foundation of the Propensity Score Matching (PSM) method, which matches individuals in the treatment and control groups based on their characteristics. The primary objective of this matching process is to minimize bias in the analysis by ensuring that individuals in the treatment group and those in the control group share similar characteristics [26, 27]. By aligning or matching propensity scores between the two groups, researchers can ensure that any differences in outcomes are more likely attributable to the treatment itself rather than to pre-existing differences between individuals. This process satisfies the Conditional Independence Assumption (CIA) [28] and enhances the accuracy and validity of research findings [25].

Accordingly, given the non-random participation in financial inclusion, we employ PSM following [25] to mitigate selection bias arising from observable characteristics. Potential bias may occur because household welfare is associated with participation in financial inclusion, while participation itself is also related to household economic conditions. Financial inclusion is treated as a binary treatment variable, and propensity scores are estimated using household socio-demographic characteristics [19]. Treated and untreated households are then matched based on these propensity scores. Under the conditional independence assumption, the estimates are interpreted as quasi-causal associations conditional on observed covariates, rather than definitive causal effects.

Methodologically, PSM was selected as the primary analytical approach given the observational and cross-sectional nature of the Susenas data and the absence of an exogenous policy rule governing access to financial services. Alternative quasi-experimental methods, such as Regression Discontinuity Design (RDD), require a clearly defined and externally imposed cutoff that deterministically assigns households into treatment and control groups [29, 30]. Such conditions are not present in the Indonesian financial inclusion context, where participation reflects household-level decisions rather than eligibility thresholds. Instrumental Variable (IV) estimation was also considered; however, its application requires a valid and strong instrument that affects financial inclusion without directly influencing household welfare [31]. The Susenas does not provide variables that plausibly satisfy the exclusion restriction, and the use of weak or invalid instruments may introduce greater bias than selection-on-observables approaches [32]. Given these constraints, PSM represents the most feasible and transparent method for reducing selection bias based on observed characteristics, allowing the estimation of quasi-causal associations conditional on observables [25, 28].

Nevertheless, an important limitation of the PSM approach is that it relies on the conditional independence assumption, which assumes that all relevant differences between treated and untreated households are captured by observable characteristics. Under this assumption, potential outcomes are independent of treatment status conditional on the included covariates [33, 34]. However, unobserved factors—such as financial literacy, cognitive functioning, risk preferences, informal social networks, or local accessibility to financial institutions—may simultaneously influence both participation in financial inclusion and household welfare, particularly among people living with disabilities. Because such factors are not fully observed in the Susenas data, the estimated associations may still be subject to hidden bias arising from unobserved confounding. To mitigate this concern, we include a rich set of household socio-demographic and economic covariates, as recommended in the matching literature [33, 35]. Nevertheless, the results should be interpreted with caution, as PSM cannot fully eliminate bias from unobserved variables. Future studies incorporating sensitivity analyses, such as Rosenbaum bounds, or more detailed data would be valuable to assess the robustness to hidden bias.

To operationalize the PSM framework, this study employs three types of variables: outcome, treatment, and covariates. The outcome variable is the average household expenditure of the household head, serving as a proxy for welfare, which includes food and non-food expenditures such as health and education spending [21]. The expenditure-based measure is preferred over income-based measures as a proxy for welfare because it more accurately reflects household living standards [19]. Consumption levels are closely linked to income and purchasing power. However, reliable income data are often difficult to obtain; therefore, an expenditure-based approach provides more valid information about actual consumption behavior [36].

Regarding treatment definition, the treatment variable is a dummy variable with the value of 1 if the household head’s financial inclusion index is ≥ 50% (classified as financially included) and 0 if it is < 50% (classified as financially excluded). The covariates include gender, age, household size, internet use, education level, employment status, residential area, types of disabilities, social assistance received (i.e. Social Rehabilitation Assistance Program/ATENSI and Family Hope Program/Program Keluarga Harapan (PKH)), and business ownership.

Procedurally, following [28], the PSM procedure consists of several steps. The first step is estimating the propensity score, which involves selecting an appropriate model and variables. Since the treatment variable in this study is binary, the probit regression model is employed to estimate the propensity score [28]. Although the logit model is also commonly used for this purpose, both models generally yield similar estimates; hence, choosing one over the other is not critical [28]. The propensity score represents the probability that an individual receives the treatment, ranging between 0 and 1 [37]. In probit regression, the propensity score is estimated by modeling the relationship between the binary treatment variable and the set of independent covariates [26]. The resulting coefficients (β) are then used to compute the propensity scores [38]. The probit model is expressed as [38]:

$$\:P({Findex}_{i}=1)\:=\:{\Phi\:}({\beta\:}_{0}+\sum\limits_{k=1}^{18}{\beta\:}_{k}{X}_{ki}+{e}_{i})$$

$\:{Findex}_{i}$=1 indicates that the household heads with disabilities is financially included (financial inclusion index ≥ 50%). The vector $\:{X}_{ki}$​ represents the set of household heads with disabilities characteristics—covering demographic factors, functional limitations, social assistance participation, and business ownership—while the coefficients $\:{\beta\:}_{k}$​ capture their marginal effects on the likelihood of being financially included. The term $\:{e}_{i}$​ denotes the error component accounting for unobserved factors.

Subsequently, the next step is selecting the matching algorithm. In this study, household heads with disabilities were identified as either treated or control units based on the ≥ 50% financial inclusion index cut-off. After computing each unit’s propensity score using the probit model, matching was conducted to pair treated and control observations with similar scores [30]. The matching algorithms applied include Nearest Neighbor (NN), Caliper and Radius, Kernel, and Local Linear matching [28].

Thereaftrer, the third step is checking the common support condition, which ensures sufficient overlap between the treatment and control groups. This step is typically verified using a density distribution graph of the propensity scores for both groups [28]. The presence of overlap indicates that the two groups share comparable propensity score ranges, allowing further analysis. Violating this assumption may lead to biased estimates because the groups differ substantially in their covariate distributions [28].

Following this, the next stage involves assessing the quality of matching. This evaluation verifies whether the matching procedure has successfully balanced the covariates between the treatment and control groups. The Standardized Bias (SB) is employed to measure differences in covariate distributions before and after matching [28]. Initially [39], suggested that an SB value of 20% indicates large bias. However, more recent studies adopt a stricter threshold of less than 5%, implying that successful matching has balanced the covariates across groups [28].

Finally, the last step is estimating the treatment effect. After verifying the matching quality, the association of financial inclusion on the welfare of people living with disabilities can be evaluated using the Average Treatment Effect on the Treated (ATT). The ATT is estimated as follows [28, 40]:

$$\:\mathrm{A}\mathrm{T}\mathrm{T}=\mathrm{E}\left({\mathrm{Y}}_{1}\right|\mathrm{D}=1)-\:\mathrm{E}{(\mathrm{Y}}_{0}|\mathrm{D}=0)$$

where:

$\:\mathrm{E}\left({\mathrm{Y}}_{1}\right|\mathrm{D}=1)$ : the mean outcome for the treatment group (financially included households, i.e., inclusion index ≥ 50%).

$\:\mathrm{E}\left({\mathrm{Y}}_{0}\right|\mathrm{D}=0)$ : the mean outcome for the control group (financially excluded households, i.e., inclusion index < 50%).

In other words, ATT represents the difference in average welfare (proxied by household expenditure) between household heads with disabilities classified as financially included and those classified as financially excluded.

## Results

### Data description

This section presents the descriptive characteristics of household heads with disabilities included in the analytical sample. The summary statistics provide an overview of the demographic, socioeconomic, and household characteristics of the study population. In terms of gender composition, 71.15% of household heads with disabilities were male. Geographically, the majority resided on Java Island (55.75%), with 53.95% living in urban areas. Regarding age distribution, following the classification by [41], 58.25% of household heads with disabilities belonged to the baby boomer generation (born between 1946 and 1964, aged 59–77 in 2023). In terms of educational attainment, 57.47% had completed only primary education or its equivalent.

From the employment perspective, 21.97% of household heads with disabilities were employed, with the majority working in the agriculture, forestry, and fisheries sectors (57.02%). In terms of household composition, 43.78% of disabled household heads had one to two household members, and 89.79% did not have any household members engaged in micro, small, and medium enterprises (MSMEs).

Lastly, regarding social assistance programs, only 0.46% of household heads with disabilities received support from the Social Rehabilitation Assistance Program (ATENSI). Meanwhile, 19.65% of them were beneficiaries of the Family Hope Program (Program Keluarga Harapan, PKH).

*Note: Highest education level is coded as a set of dummy variables (i.lasteduc), with no schooling used as the reference category and therefore not reported. Employment status is coded as a set of dummy variables (i.jobstatus), with working/employed used as the reference category and therefore not reported.

Table 1 presents the descriptive statistics of the main research variables used in this study. As shown in Table 1, the average monthly household expenditure of heads of households with disabilities, expressed in natural logarithmic form (ln), is 14.89 (equivalent to USD 191.44) with a standard deviation of 0.71. Following best practice in statistics, the logarithmic transformation is applied to reduce the skewness of expenditure data and to mitigate the influence of extreme values commonly observed in household consumption variables [42]. This practice is also consistent with recent empirical studies employing propensity score matching (PSM) that use household expenditure as an outcome variable [18, 19]. The relatively small deviation indicates that variations in household expenditure among households with disabilities are not highly dispersed, suggesting that most households have expenditures close to the mean value. Meanwhile, the minimum log expenditure is 11.73 (equivalent to USD 8.12) and the maximum log expenditure is 18.84 (equivalent to USD 9941) per month, indicating a substantial expenditure gap between households categorized as poor and those categorized as wealthy.

**Table 1:** Descriptive statistics of research variables

Variable	Obs	Mean	Std. Dev.	Min	Max
Average monthly household expenditure of heads of households with disabilities (lnexpend)	44,107	14.89	0.71	11.73	18.84
Financial inclusion index (1 = financially included, if financial inclusion index ≥ 50%; 0 = financially excluded, if index < 50%) (fininc_stat_krt_50)	44,107	0.26	0.44	0	1
Gender (1 = male; 0 = female)	44,107	0.68	0.46	0	1
Age (years)	44,107	62.89	11.92	14	97
Household size (hhsize)	44,107	2.86	1.68	1	18
Internet use (1 = yes; 0 = no)	44,107	0.25	0.43	0	1
*Highest education level (i.lasteduc)					
No/Not completed primary education	44,107	0.25	0.44	0	1
Primary education	44,107	0.32	0.47	0	1
Junior secondary education	44,107	0.12	0.32	0	1
Senior secondary education	44,107	0.15	0.35	0	1
Tertiary education	44,107	0.06	0.23	0	1
*Employment status (i.jobstatus)					
Attending school	44,107	0.00	0.01	0	1
Managing household	44,107	0.27	0.44	0	1
Other activities	44,107	0.37	0.48	0	1
Not working	44,107	0.12	0.33	0	1
Residential area (1 = urban; 0 = rural) (resarea)	44,107	0.37	0.48	0	1
Difficulty/impairment in seeing (seeingdiff)	44,107	0.67	0.47	0	1
Difficulty/impairment in hearing (hearingdiff)	44,107	0.31	0.46	0	1
Difficulty/impairment in walking or climbing stairs (walkinglimit)	44,107	0.41	0.49	0	1
Difficulty/impairment in using or moving hands/fingers (handmovelimit)	44,107	0.15	0.36	0	1
Difficulty/impairment in remembering or concentrating (rememberdiff)	44,107	0.24	0.43	0	1
Behavioral and/or emotional impairments (behaveimpair)	44,107	0.08	0.27	0	1
Difficulty/impairment in speaking or understanding/communicating with others (speakingdiff)	44,107	0.10	0.30	0	1
Difficulty/impairment in self-care (selfcarediff)	44,107	0.10	0.30	0	1
Social Rehabilitation Assistance (ATENSI) (1 = recipient; 0 = non-recipient)	44,107	0.006	0.08	0	1
Family Hope Program (PKH) (1 = recipient; 0 = non-recipient)	44,107	0.19	0.39	0	1
Business ownership (1 = yes; 0 = no)	44,107	0.11	0.31	0	1

Highest education level is coded as a set of dummy variables (i.lasteduc), with no schooling used as the reference category and therefore not reported. Employment status is coded as a set of dummy variables (i.jobstatus), with working/employed used as the reference category and therefore not reported

Furthermore, the average financial inclusion index is 0.2639, indicating that 26.39% of household heads with disabilities are categorized as financially included. The standard deviation of 0.44 suggests a considerable disparity between households that are financially included and those that remain financially excluded. The minimum and maximum values of 0 and 1, respectively, reflect the binary nature of the variable, consistent with the dummy classification applied in the model.

For ordinal variables such as the highest education level, interpretation is based on the distribution of respondents across education categories rather than on the mean value. As shown in the descriptive statistics, the educational attainment of heads of households with disabilities is concentrated at lower levels: approximately 25.5% have no formal schooling, 32.2% have completed primary education, and 11.9% have completed junior secondary education. In contrast, only 14.5% have completed senior secondary education and 5.8% have attained tertiary education.

### Financial inclusion index calculation results

The first stage prior to conducting further analysis involved calculating the financial inclusion index, which serves as the treatment variable in this study. Table 2 presents the distribution of the financial inclusion index based on the number of financial inclusion indicators used by heads of households with disabilities.

**Table 2:** Financial inclusion index calculation results

Number of Financial Inclusion Indicators	Freq.	Percent	Cum.
0	4,036,526	46.26	46.26
1	2,436,439	27.92	74.18
2	1,324,888	15.18	89.36
3	775,276	8.88	98.25
4	152,974	1.75	100
Total	8,726,103	100	

As shown in Table 2, a score of 0 indicates that heads of households with disabilities did not use any financial inclusion indicators, representing 46.26% of the total weighted sample from the Susenas dataset used in this study. A score of 1 indicates that the household head used one out of four financial inclusion indicators, accounting for 27.92% of the sample. A score of 2 indicates the use of two indicators, representing 15.18%. A score of 3 indicates the use of three indicators, comprising 8.88%, while a score of 4 indicates that the household head used all four financial inclusion indicators, accounting for 1.75% of the sample. In other words, the majority of heads of households with disabilities still have limited access to financial institutions, and only a small proportion utilize a diverse range of financial services.

To classify household heads with disabilities as either financially included or financially excluded, a binary transformation of the financial inclusion index was applied using a cut-off point of ≥ 50% (equivalent to the use of at least two weighted indicators). This threshold follows the approach of [14, 15, 19]. Table 3 reports the distribution of financial inclusion status using alternative cut-off points of ≥ 50%, ≥ 25%, and ≥ 75%.

**Table 3:** Financial inclusion index by different cut-off points

Financial Inclusion	Cut-off ≥ 50%		Cut-off ≥ 25%	Cut-off ≥ 75%			
Status of Household Heads with Disabilities	Freq.	%	Freq.	%	Freq.	%	
Financially Excluded	6,472,965	74.18	4,036,526	46.26	7,797,853	89.36	
Financially Included	2,253,138	25.82	4,689,577	53.74	928,250	10.64	
Total	8,726,103	100	8,726,103	100	8,726,103	100	

As shown in Table 3, under the ≥ 50% cut-off point, the financial inclusion index is represented as a dummy variable, where 1 = financially included (financial inclusion index ≥ 50%) and 0 = financially excluded (financial inclusion index < 50%). Financially included households [1] are those in which the head of household uses at least two out of four financial inclusion indicators, corresponding to the sum of categories 2, 3, and 4, totaling 2,253,138 individuals (25.82%). Conversely, financially excluded households (0) are those using fewer than two indicators, corresponding to categories 0 and 1, totaling 6,472,965 individuals (74.18%). To address concerns regarding potential oversimplification of this multidimensional construct and to assess the robustness of the findings, this study further examines the association of financial inclusion on the welfare of people living with disabilities using alternative cut-off points of ≥ 25% and ≥ 75%.

The financial inclusion index with a cut-off of ≥ 25% is also represented as a dummy variable, where 1 = financially included (financial inclusion index ≥ 25%) and 0 = financially excluded (financial inclusion index < 25%). In this case, financially included households [1] are those whose heads use at least one out of four financial inclusion indicators, comprising categories 1, 2, 3, and 4, totaling 4,689,577 individuals (53.74%). Meanwhile, financially excluded households (0) refer to those that did not use any of the four indicators, totaling 4,036,526 individuals (46.26%).

The financial inclusion index with a cut-off of ≥ 75% is defined similarly as a dummy variable, where 1 = financially included (financial inclusion index ≥ 75%) and 0 = financially excluded (financial inclusion index < 75%). Accordingly, as shown in Table 3, financially included households [1] are those in which the head of household uses at least three out of four financial inclusion indicators, corresponding to categories 3 and 4, totaling 928,250 individuals (10.64%). In contrast, financially excluded households (0) are those using fewer than three indicators, corresponding to categories 0, 1, and 2, totaling 7,797,853 individuals (89.36%).

### PSM results

After calculating the financial inclusion index, which serves as the treatment variable, the subsequent stage involved estimating the PSM model. Propensity scores were generated using a probit regression framework, with financial inclusion status as the dependent variable and a set of socioeconomic, demographic, and disability-related characteristics as covariates. Table 4 reports the probit regression results used to estimate the propensity scores under three alternative cut-off points of the financial inclusion index (≥ 50%, ≥ 25%, and ≥ 75%).

**Table 4:** Probit regression output

Variables	Cut-off$\:\ge\:$50% of the financial inclusion index	Cut-off$\:\ge\:$25% of the financial inclusion index	Cut-off$\:\ge\:$75% of the financial inclusion index
gender	-0.05***	-0.09***	-0.002
	(0.005)	(0.005)	(0.002)
age	0.001	0.007***	0.002***
	(0.001)	(0.001)	(0.00)
agesq	-3.70	-4.71***	-1.83***
	(1.09)	(1.29)	(6.06)
hhsize	0.003**	-0.001	0.005***
	(0.001)	(0.001)	(0.00)
internet	0.21***	0.26***	0.08***
	(0.005)	(0.006)	(0.002)
2.lasteduc	0.03***	0.04***	0.01***
	(0.007)	(0.009)	(0.003)
3.lasteduc	0.08***	0.08***	0.03***
	(0.007)	(0.009)	(0.003)
4.lasteduc	0.14***	0.15***	0.07***
	(0.009)	(0.01)	(0.005)
5.lasteduc	0.26***	0.27***	0.13***
	(0.008)	(0.01)	(0.006)
6.lasteduc	0.51***	0.41***	0.30***
	(0.01)	(0.01)	(0.01)
2.jobstatus	-	-	-
3.jobstatus	0.01*	-0.02**	0.003
	(0.006)	(0.008)	(0.003)
4.jobstatus	0.02***	-0.003	0.01***
	(0.006)	(0.007)	(0.003)
5.jobstatus	-0.01*	-0.03***	0.004
	(0.008)	(0.009)	(0.005)
resarea	0.04***	0.06***	0.02***
	(0.004)	(0.005)	(0.002)
seeingdiff	0.003	-0.01**	0.00
	(0.004)	(0.005)	(0.002)
hearingdiff	-0.02***	-0.03***	-0.008***
	(0.005)	(0.005)	(0.002)
walkinglimit	-0.002	-0.01**	-0.00
	(0.005)	(0.005)	(0.002)
handmovelimit	-0.004	0.007	-0.005
	(0.007)	(0.008)	(0.003)
rememberdiff	-0.002	-0.008	0.007**
	(0.005)	(0.006)	(0.003)
behaveimpair	-0.01	-0.002	0.003
	(0.008)	(0.01)	(0.005)
speakingdiff	-0.04***	-0.04***	-0.03***
	(0.008)	(0.009)	(0.005)
selfcarediff	-0.00	-0.009	0.006
	(0.01)	(0.009)	(0.004)
atensi	-0.03	0.05	-0.02
	(0.03)	(0.03)	(0.02)
pkh	0.01**	0.17***	-0.05***
	(0.005)	(0.006)	(0.003)
business	0.04***	0.02***	0.02***
	(0.006)	(0.007)	(0.004)
Observations	44,104	44,104	44,104

*lasteduc* is specified as a set of dummy variables. Category 1 (no schooling/never attended school) serves as the reference group. The coding is: 2 did not complete primary school, 3 = primary school or equivalent, 4 = junior secondary school or equivalent, 5 = Senior secondary school/vocational high school or equivalent, and 6 = tertiary education.

*jobstatus* is specified as a set of dummy variables. Category 1 (working) serves as the reference group. The coding is: 2 = attending school, 3 = household duties, 4 = other non-personal activities, and 5 = not engaged in any activity.

The coefficient for *jobstatus* is not reported because this category contains no valid observations. In the sample, three household heads were identified as still pursuing their education, resulting in an effective sample size of 44,104 observations across all model specifications.

Robust standard errors in parentheses ***p<0.01, **p<0.05, *p<0.1

As shown in Table 4, based on the marginal effects of the probit regression indicate that several socioeconomic factors—including gender, age, household size, internet usage, education level, employment status, residential area, participation in the Family Hope Program (PKH), and business ownership—were found to have a statistically significant influence on the likelihood of being financially included mostly at the 1% significance level (*p* < 0.01) across all cut-off points.

Meanwhile, among the disability-related variables, only hearing impairment and speech/communication difficulties demonstrated a consistently significant negative effect on financial inclusion at *p* < 0.01 across all cut-off points. In contrast, other types of disabilities—such as visual impairment, walking limitations, and memory or concentration difficulties—showed a moderate and less consistent effect, with statistical significance at *p* < 0.05 for the ≥ 25% and ≥ 75% cut-offs. These findings suggest that while socioeconomic characteristics remain the most robust predictors of financial inclusion, disability types—particularly those affecting sensory and communication abilities—continue to serve as structural barriers limiting access to and participation in formal financial systems among people with disabilities in Indonesia.

After estimating the propensity scores, the next step was to perform matching between units in the treatment and control groups. As shown in the Supplementary File 1 (Appendix 2), before matching (unmatched), all covariate variables exhibited imbalances between the treatment and control groups, as indicated by the magnitude of the bias and the significance of the t-test values. However, after matching, the bias values for all covariates substantially decreased, and most p-values from the t-tests became statistically insignificant. This indicates that the differences in characteristics between the treatment and control groups were successfully reduced after matching.

Following the matching procedure, a common support test (Supplementary File 1, Appendix 3) was conducted to ensure sufficient overlap between the treatment and control groups using the density distribution analysis of the propensity scores [28]. The figure above illustrates that, in the left panel (raw/before matching), there was already an observable overlap between the treated (financially included) and control (financially excluded) groups, although the propensity score distributions were not yet balanced. After matching, as shown in the right panel (matched), the propensity score distributions of both groups became similar and balanced, indicating that the common support assumption of the PSM method was satisfied.

The next stage involved evaluating the quality of the matching to ensure that the treatment and control groups were comparable after the matching process. The main indicator used was the mean bias value [28]. suggest that although there is no universally accepted threshold for assessing matching quality using mean bias, many empirical studies consider a value below 5% as an indication of successful covariate balance. Based on the results presented in Supplementary File 1 (Appendix 4), the mean bias values for the ≥ 50% and ≥ 25% cut-offs are both below 5%, indicating that the matching successfully balanced the covariates between the treatment and control groups. Meanwhile, for the ≥ 75% cut-off, the mean bias values are slightly higher but remain below 20%, implying that the bias is not large [39].

After completing all the PSM procedures described above, the association of financial inclusion on the welfare of people living with disabilities was estimated using the ATT [28]. Table 5 reports the ATT estimates obtained from four different matching algorithms—Kernel, Nearest Neighbour, Radius, and Local Linear—under three alternative cut-off points of the financial inclusion index (≥ 50%, ≥ 25%, and ≥ 75%).

**Table 5:** Average Treatment effect on the Treated (ATT)

Cut-off	Matching Algorithm	Difference	S.E.	T-stat
$\:\ge\:$50%	Kernel	0.2947***	0.0099	29.72
	Nearest Neighbour	0.2949***	0.0128	23.13
	Radius	0.2976***	0.0099	30.18
	Local linear	0.2987***	0.0128	23.42
$\:\ge\:$25%	Local linear	0.2283***	0.0109	20.83
	Nearest Neighbour	0.2285***	0.0109	20.85
	Kernel	0.2298***	0.0084	27.20
	Radius	0.2343***	0.0079	29.31
$\:\ge\:$75%	Kernel	0.3123***	0.0122	25.60
	Nearest Neighbour	0.3147***	0.0167	18.82
	Local linear	0.3201***	0.0167	19.15
	Radius	0.3323***	0.0122	27.34

Notes: *** *p* < 0.01, ** *p* < 0.05, * *p* < 0.1

As shown in Table 5, financial inclusion is consistently associated with higher household welfare across all matching algorithms and cut-off points. At the ≥ 50% cut-off, the ATT values obtained from the Nearest Neighbour to Radius matching techniques range between 0.2947 and 0.2987, indicating that households headed by people living with disabilities who have access to at least two financial inclusion indicators have 29.47%-29.87% higher welfare levels compared to those with access to fewer than two indicators, after controlling for observable differences between the two groups (*p* < 0.01).

At the ≥ 25% cut-off, the ATT values from the Local Linear to Radius matching techniques range between 0.2283 and 0.2343, implying that households headed by people living with disabilities who have access to at least one financial inclusion indicator exhibit 22.83%-23.43% higher welfare levels compared to those without access to any of the four financial inclusion indicators, after controlling for observable differences (*p* < 0.01). Lastly, at the ≥ 75% cut-off, the ATT values from the Nearest Neighbour to Radius matching techniques range between 0.3123 and 0.3323, showing that households headed by people living with disabilities who have access to at least three financial inclusion indicators have 31.23%-33.23% higher welfare levels compared to those with access to fewer than three indicators, after controlling for covariate differences between the two groups (*p* < 0.01).

## Discussion

This study aims to analyze the quasi-causal association between financial inclusion and the welfare of people living with disabilities in Indonesia using the March 2023 wave of the Susenas. Based on the estimation results obtained through the PSM method, the ATT values across three different cut-off levels are all positively and significantly associated with various welfare levels of households headed by people living with disabilities. These results indicate that the higher the level of financial inclusion among household heads with disabilities, the greater their level of welfare. The results underscore the critical function of financial inclusion as an enabler of socioeconomic empowerment, especially for marginalized populations. Access to formal financial services such as savings, credit, and insurance enables households to smooth consumption, mitigate income shocks, and invest in human and productive capital [43]. For people living with disabilities—who often encounter systemic barriers in employment, education, and social participation—financial inclusion serves not only as an economic instrument but also as a mechanism for social inclusion [44]. Thus, improved access to financial services helps reduce multidimensional poverty and strengthens resilience among people with disabilities, who are disproportionately affected by economic vulnerabilities.

However, the magnitude of the estimated welfare gains should be interpreted with caution. The observed associations—ranging from approximately 23% to 33% across different inclusion thresholds—are economically meaningful but may not be attributable solely to financial inclusion itself, particularly given the structural constraints faced by households headed by people living with disabilities. In this context, financial inclusion is better understood as a gateway or enabling mechanism rather than a standalone solution. While access to financial services may facilitate welfare-enhancing behaviors, its full potential is likely to be realized only when complemented by broader disability-inclusive policies to accommodate the specific needs of people living with disabilities. Moreover, while the binary specification of the financial inclusion index facilitates implementation within the PSM framework, it may not fully capture heterogeneity in the composition and depth of financial inclusion. Nevertheless, the use of multiple cut-off thresholds in this study partially addresses this concern by demonstrating consistent associations across varying levels of inclusion intensity.

Specifically, the main analysis, which uses a cut-off of ≥ 50%, shows that financially included households—those whose heads use at least two out of four financial inclusion indicators—enjoy higher welfare than financially excluded ones. When applying the alternative thresholds of ≥ 25% and ≥ 75%, the positive association between financial inclusion and welfare remains evident, although the association magnitude slightly differs. This pattern suggests that even a lower level of financial inclusion (≥ 25%) already contributes meaningfully to welfare improvement, while deeper inclusion (≥ 75%) tends to amplify the benefits for those who can sustain greater financial engagement. Such findings are in line with Iddrisu and Danquah [15] who found that incremental increases in financial access significantly improve household welfare in Ghana, and with Abokyi and Bettin [18], who observed that financial inclusion enhances economic well-being by facilitating productive investments and consumption smoothing.

Consistent with these results, the findings of this study align with prior research reporting a positive link between financial inclusion and household welfare [16, 17, 19]. However, beyond reinforcing existing evidence, this study extends the literature by specifically focusing on people living with disabilities—an often-overlooked demographic in the financial inclusion discourse in Indonesia and Southeast Asia—and by examining the relationship across multiple inclusion thresholds. This broader analytical approach provides a more nuanced understanding of how varying degrees of inclusion affect welfare outcomes among people living with disabilities, thereby complementing the general population-focused evidence from previous studies.

At the same time, while the overall association of financial inclusion is positive, it is important to note that this study examines welfare primarily from a financial perspective and does not capture broader dimensions of well-being, such as health status, social participation, or subjective quality of life. Existing research cautions that access alone may not fully close welfare gaps between people with and without disabilities [45], for example, found that people living with disabilities continue to experience lower financial well-being than their non-disabled peers due to persistent challenges in employment and accessibility to financial products. Similarly [46], demonstrated that the benefits of financial inclusion are not uniform across all expenditure categories—showing a stronger association on education spending than on food or health expenditure. These findings imply that although financial inclusion supports welfare improvement, complementary policies—such as targeted vocational programs, accessible fintech services, and tailored financial literacy initiatives—are essential to ensure that people with disabilities can fully capitalize on available financial opportunities.

In addition, this study has several limitations that should be acknowledged. First, due to the cross-sectional nature of the data, the analysis cannot fully rule out the possibility of reverse causality. While financial inclusion may be associated with higher household welfare, it is also plausible that households with higher welfare levels are more likely to access and utilize formal financial services. This concern is particularly salient given that household expenditure is used as a proxy for welfare. Consequently, the estimated effects should be interpreted as quasi-causal associations conditional on observed characteristics, rather than definitive evidence of temporal or causal relationships. Future research employing longitudinal data or quasi-experimental designs would be required to more clearly establish the direction and timing of the relationship between financial inclusion and household welfare.

In the Indonesian context, the findings of this study demonstrate that financial inclusion is positively and significantly associated with the welfare of people living with disabilities. This association is particularly relevant given that Indonesia has established several policy frameworks aimed at guaranteeing equal opportunities for people living with disabilities across various aspects of national and social life [6, 7, 10]. Nevertheless, in practice, people living with disabilities continue to face substantial challenges in accessing financial services, indicating that disability-inclusive financial inclusion has yet to be fully realized. The most commonly reported barriers include limited physical accessibility to financial institutions and communication constraints during service interactions [5].

Moreover, people living with disabilities experience structural inequalities in accessing goods and services, including those in the financial sector. These inequalities are partly driven by the additional costs required to access services that are more readily available to non-disabled individuals. Such extra costs contribute to persistent disparities in bank account ownership, access to digital financial services, and access to credit from formal financial institutions, all of which remain significantly lower among people living with disabilities than among their non-disabled counterparts. These disparities are further compounded by demographic factors. For instance, bank account ownership among people living with disabilities is disproportionately concentrated among individuals aged over 50 years, whereas non-disabled individuals are more likely to hold bank accounts during their economically active ages of 15–50 years. Moreover, geographic inequalities exacerbate these gaps, as financial inclusion among people living with disabilities is largely concentrated in urban areas and on the island of Java, reflecting uneven regional development and access to financial infrastructure across Indonesia [5].

Even so, encouraging developments have emerged, particularly through corporate commitments to Environmental, Social, and Governance (ESG) principles. Such commitments encourage companies to develop strategic plans that ensure the availability of inclusive financial services for people living with disabilities. This initiative is particularly important because people living with disabilities possess significant economic potential and can broaden the customer base. Beyond the social benefits, disability-inclusive financial approaches may also generate positive spillover effects for non-disabled consumers and enhance corporate reputation among investors [5].

Taken together, these findings highlight two important considerations for strengthening disability-inclusive financial inclusion in Indonesia. First, the availability of valid, high-quality, and comprehensive data on people living with disabilities is crucial to support targeted and evidence-based policy design. The absence of an integrated national disability database limits the government’s ability to accurately identify needs, monitor program effectiveness, and formulate precise interventions. Second, advancing meaningful financial inclusion for people living with disabilities requires cross-sectoral collaboration involving government agencies, financial service providers, disability organizations, the broader community, and technological innovators. Such coordinated efforts are essential to ensure that financial products and delivery channels are accessible, adaptive, and responsive to the unique circumstances faced by people living with disabilities. In this regard, Indonesia could draw lessons from international experiences, such as those of the United Kingdom, Australia, and India, where more developed disability data systems and cross-sectoral collaboration have been emphasized in advancing disability-inclusive policies.

## Conclusions

This study provides robust empirical evidence that financial inclusion is positively and significantly associated with the welfare of people living with disabilities in Indonesia. Using Propensity Score Matching (PSM) and multiple cut-off thresholds, the findings consistently show that households headed by people living with disabilities who are financially included experience substantially higher welfare levels than their financially excluded counterparts, with estimated gains ranging from approximately 23% for households with minimal financial access to over 33% for those with deeper financial engagement. These results underscore that both access to and the intensity of financial inclusion matter for improving socioeconomic well-being. From a policy perspective, financial inclusion should therefore be recognized as an important enabling mechanism within Indonesia’s disability-inclusive development agenda, although it cannot operate effectively in isolation. Its welfare-enhancing potential depends on complementary interventions that address structural barriers such as accessibility constraints, demographic and regional inequalities, and additional costs faced by people living with disabilities. Accordingly, strengthening disability-disaggregated data systems and fostering cross-sectoral collaboration among government agencies, financial service providers, disability organizations, and technological innovators are essential to ensure that financial inclusion translates into sustainable welfare improvements for people living with disabilities in Indonesia.

## Supplementary Information

Supplementary Material 1.

## Data Availability

All data is publicly accessible upon formal request through the official Statistics Indonesia (Badan Pusat Statistik/BPS) data portal at (https://silastik.bps.go.id/v3/index.php/site/login/).

## Abbreviations

ATENSI: Social Rehabilitation Assistance Program
ATT: Average Treatment Effect on the Treated
PKH: Family Hope Program
PSM: Propensity Score Matching
SNKI: National Strategy for Financial Inclusion
Susenas: National Socio-Economic Survey
