# Supplementary material for: Does financial inclusion improve the welfare of people living with disabilities? evidence from Indonesia using a propensity score matching approach
Source: BMC Public Health. 2026 Mar 20;26:1394. doi: 10.1186/s12889-026-27080-2 (PMC13126807; doi:10.1186/s12889-026-27080-2)
Supplement: Supplementary file 1 — Supplementary Material 1. [file 12889_2026_27080_MOESM1_ESM.docx]

**Appendix 1** Definition of social assistance received by people living with disabilities

1. Social Rehabilitation Assistance Program/ATENSI refers to a social rehabilitation service that adopts a family-based, community-based, and/or residential approach through activities such as support for meeting adequate living needs, social care and/or child caregiving, family support, physical therapy, psychosocial therapy, spiritual-mental therapy, vocational training and entrepreneurship development, social assistance and facilitation, as well as accessibility support.
2. The Family Hope Program/Program Keluarga Harapan (PKH) is a conditional cash transfer program provided to low-income households listed in the Integrated Social Welfare Data (DTKS) and meeting the eligibility criteria established for PKH beneficiaries, including households with members who have severe disabilities.

**Appendix 2** Covariate balance output

| Variables | Unmatched | Mean | | %reduc | | t-test | |
| --- | --- | --- | --- | --- | --- | --- | --- |
|  | Matched | Treated | Control | %bias | \|bias\| | t | p>\|t\| |
| gender | U | 0.68 | 0.68 | 5.0 |  | 4.64 | 0.000 |
|  | M | 0.69 | 0.70 | 0.1 | 81.2 | 0.73 | 0.466 |
| age | U | 60.65 | 63.69 | -25.5 |  | -23.83 | 0.000 |
|  | M | 60.65 | 60.04 | 5.1 | 80.0 | 3.85 | 0.000 |
| hhsize | U | 3.02 | 2.81 | 12.5 |  | 11.56 | 0.000 |
|  | M | 3.02 | 3.01 | 0.4 | 96.8 | 0.30 | 0.763 |
| internet | U | 0.49 | 0.16 | 74.3 |  | 74.18 | 0.000 |
|  | M | 0.49 | 0.48 | 1.4 | 98.2 | 0.91 | 0.365 |
| 2.lasteduc | U | 0.15 | 0.29 | -36.4 |  | -31.87 | 0.000 |
|  | M | 0.15 | 0.15 | -1.4 | 96.1 | -18.99 | 0.210 |
| 3.lasteduc | U | 0.25 | 0.35 | -21.0 |  | -0.14 | 0.000 |
|  | M | 0.25 | 0.25 | -0.2 | 99.2 | 6.41 | 0.892 |
| 4.lasteduc | U | 0.14 | 0.11 | 6.8 |  | 0.58 | 0.000 |
|  | M | 0.14 | 0.13 | 0.8 | 88.5 | 0.49 | 0.564 |
| 5.lasteduc | U | 0.26 | 0.10 | 42.1 |  | 42.71 | 0.000 |
|  | M | 0.26 | 0.25 | 3.7 | 91.2 | 2.45 | 0.014 |
| 6.lasteduc | U | 0.16 | 0.02 | 50.2 |  | 57.64 | 0.000 |
|  | M | 0.16 | 0.17 | -4.5 | 91.0 | -2.57 | 0.010 |
| 2.jobstatus | U | 0.003 | 0 | 2.3 |  | 2.89 | 0.004 |
|  | M | 0 | 0 | 0.0 | 100 | . | . |
| 3.jobstatus | U | 0.27 | 0.27 | -0.6 |  | -0.58 | 0.565 |
|  | M | 0.27 | 0.27 | -0.4 | 37.7 | -0.30 | 0.767 |
| 4.jobstatus | U | 0.40 | 0.35 | 10.3 |  | 9.64 | 0.000 |
|  | M | 0.40 | 0.40 | -0.1 | 99.5 | -0.04 | 0.968 |
| 5.jobstatus | U | 0.09 | 0.13 | -11.7 |  | -10.46 | 0.000 |
|  | M | 0.09 | 0.09 | 2.0 | 83.2 | 1.64 | 0.101 |
| resarea | U | 0.49 | 0.32 | 34.6 |  | 32.55 | 0.000 |
|  | M | 0.49 | 0.47 | 4.1 | 88.1 | 3.06 | 0.002 |
| seeingdiff | U | 0.67 | 0.68 | -1.7 |  | -1.56 | 0.118 |
|  | M | 0.67 | 0.68 | -1.4 | 19.6 | -1.03 | 0.302 |
| hearingdiff | U | 0.24 | 0.34 | -21.3 |  | -19.30 | 0.000 |
|  | M | 0.24 | 0.23 | 2.6 | 87.8 | 2.11 | 0.035 |
| walkinglimit | U | 0.38 | 0.43 | -9.6 |  | -8.83 | 0.000 |
|  | M | 0.38 | 0.38 | 0.9 | 90.3 | 0.72 | 0.473 |
| handmovelimit | U | 0.14 | 0.16 | -5.8 |  | -5.26 | 0.000 |
|  | M | 0.14 | 0.13 | 2.3 | 59.4 | 1.86 | 0.062 |
| rememberdiff | U | 0.20 | 0.26 | -13.9 |  | -12.58 | 0.000 |
|  | M | 0.20 | 0.19 | 0.9 | 93.8 | 0.69 | 0.490 |
| behaveimpair | U | 0.07 | 0.09 | -5.3 |  | -4.85 | 0.000 |
|  | M | 0.07 | 0.07 | -0.1 | 98.2 | -0.08 | 0.938 |
| speakingdiff | U | 0.07 | 0.11 | -15.3 |  | -13.52 | 0.000 |
|  | M | 0.07 | 0.07 | 0.8 | 95.0 | 0.67 | 0.502 |
| selfcarediff | U | 0.09 | 0.11 | -6.9 |  | -6.22 | 0.000 |
|  | M | 0.09 | 0.08 | 2.0 | 70.6 | 1.64 | 0.100 |
| atensi | U | 0.004 | 0.007 | -3.0 |  | -2.63 | 0.008 |
|  | M | 0.004 | 0.005 | -0.7 | 76.7 | -0.57 | 0.566 |
| pkh | U | 0.16 | 0.21 | -13.4 |  | -12.09 | 0.000 |
|  | M | 0.16 | 0.17 | -2.8 | 78.9 | -2.26 | 0.024 |
| business | U | 0.13 | 0.10 | 9.2 |  | 8.78 | 0.000 |
|  | M | 0.13 | 0.13 | 1.5 | 83.8 | 1.09 | 0.274 |

**Appendix 3** Common support test output

**
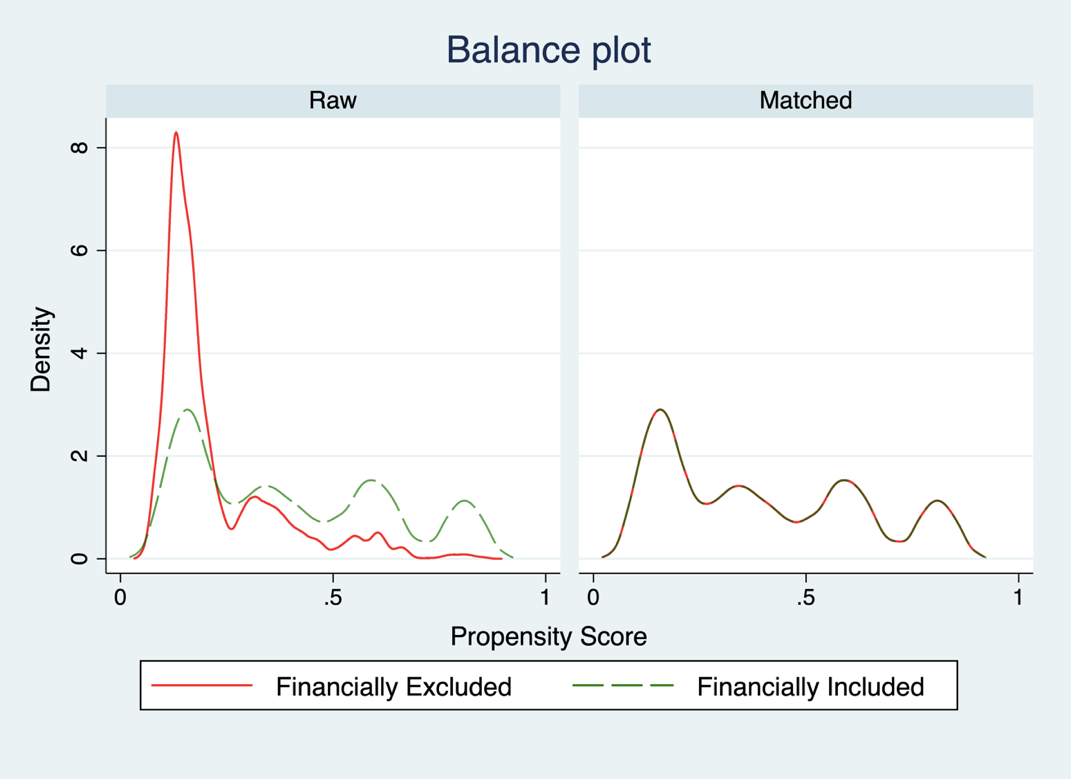
**

**Appendix 4** Matching quality evaluation output

| Cut-off | Matching algorithm | p>chi2 | Mean bias |
| --- | --- | --- | --- |
| $\geq$50% | Nearest Neighbour | 0.000 | 1.6 |
|  | Kernel | 0.001 | 1.4 |
|  | Radius | 0.000 | 2.0 |
|  | Local linear | 0.000 | 1.6 |
| $\geq$25% | Nearest Neighbour | 0.000 | 2.2 |
|  | Kernel | 0.000 | 1.6 |
|  | Radius | 0.000 | 1.8 |
|  | Local linear | 0.000 | 2.2 |
| $\geq$75% | Nearest Neighbour | 0.000 | 8.9 |
|  | Kernel | 0.000 | 8.0 |
|  | Radius | 0.000 | 6.4 |
|  | Local linear | 0.000 | 8.9 |
